# Biokinetic datasets of PEI F25-LMW complexed and non-complexed ^32^P-siRNA within different lung compartments

**DOI:** 10.1016/j.dib.2016.03.092

**Published:** 2016-04-01

**Authors:** Jens Lipka, Manuela Semmler-Behnke, Alexander Wenk, Jana Burkhardt, Achim Aigner, Wolfgang Kreyling

**Affiliations:** a Comprehensive Pneumology Center Institute of Lung Biology and Disease, Helmholtz Zentrum München – German Research Center for Environmental Health, Ingolstaedter Landstraße 1, 85764 Neuherberg, Germany; b Philipps-University of Marburg Department of Pharmaceutics and Biopharmacy, Ketzerbach 63, 35037 Marburg, Germany; c Fraunhofer Institute for Cell Therapy and Immunology (IZI) Leipzig, Perlickstraße 1, 04103 Leipzig, Germany; d Rudolf-Boehm-Institute for Pharmacology and Toxicology, Clinical Pharmacology University of Leipzig, Haertelstrasse 16–18, 04107 Leipzig, Germany

**Keywords:** Biokinetic data, Lung, PEI

## Abstract

Biokinetics data of lung-administered *PEI F25-LMW/siRNA polyplexes within different lung compartments are presented. Thereby, at three different timepoints* (1 h, 3 h, 8 h), *the data was determined by calculations* to the ^32^P-radioactivity in the whole mouse body. Additionally, data was optimized to the available *PEI F25-LMW/siRNA polyplexes in the target organ and therefore normalized* to the sum of all lung compartments. Methods, other biokinetics data and the discussion of the results are published in “*Biokinetic studies of non-complexed siRNA versus nano-sized PEI F25-LMW/siRNA polyplexes following intratracheal instillation into mice*” (Lipka et al., 2016 [1]).

**Specifications Table**

**Table:** 

Subject area	*Pharmacy*
More specific subject area	*Biopharmacy of nano-sized polyplexes*
Type of data	*Figure*
How data was acquired	*Liquid scintillation counting (LSC), TriCarb 2500 liquid scintillation counter (Perkin Elmer, Rodgau, Germany)*
Data format	*Analyzed*
Experimental factors	*Lung samples were harvested at three different time points*
Experimental features	*Lungs were rinsed, liquid was separated from the cells, all samples treated with nitric acid,*^32^*P-siRNA measured by LSC*
Data source location	*Neuherberg (Munich), Germany*
Data accessibility	*Data are presented in this article*

**Value of the data**

• Data gives a quick overview of the distribution of PEI F25-LMW/^32^P-siRNA nanoscale complexes (polyplexes) and non-complexed ^32^P-siRNA within the lungs.
• Data serve as one potential risk assessment factor for polyplexes of the same / similar size that are supposed to be applied to the lungs.
• Data serve as a comparison value to other nano-sized spheres either in regard to the applied dose (total animal) or in regard to the available dose in the target organ (lungs).

## 1 Data

**Fig. 1:**
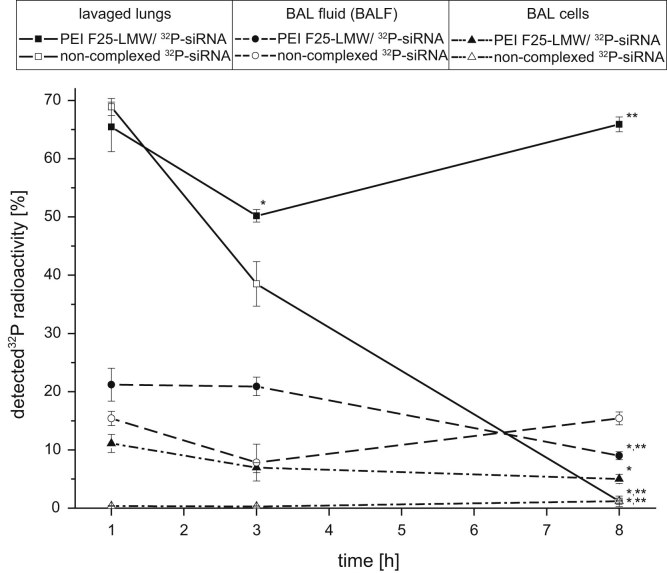
Kinetic pattern of ^32^P-siRNA versus PEI F25-LMW/^32^P-siRNA polyplexes in BAL/lung compartments after i.t. instillation into mice [2]. Values are given in mean±SEM (*n*≥3). *Significantly different to the 1 h value. § – Significantly different to the 3 h value.

**Fig. 2:**
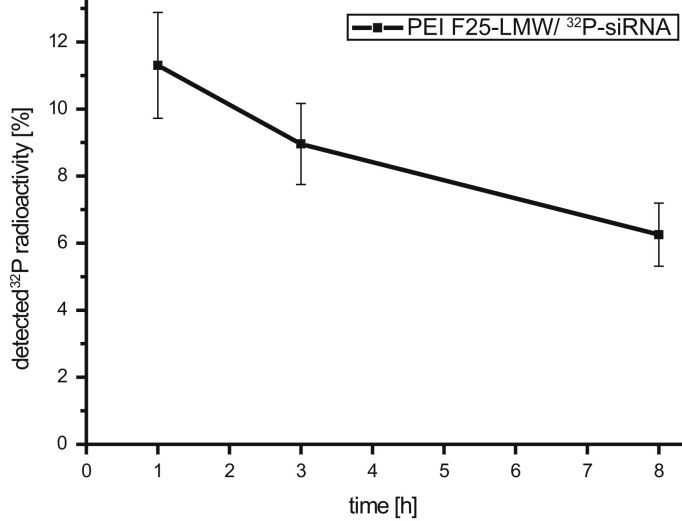
Kinetic pattern of PEI F25-LMW/^32^P-siRNA polyplexes in BAL cells calculated relative to the total lung ^32^P-activity. Values are given in mean±SEM (*n*≥3).

The diagram of Fig. 1 shows the biokinetics (measured ^32^P-radioactivity) of non-complexed ^32^P-siRNA and PEI F25-LMW complexed ^32^P-siRNA within different lung compartments after intratracheal instillation. Data points were relatively calculated to the radioactivity in the whole mouse body. While only limited data is available in the literature, the second figure focuses on the uptake by broncho alveolar (BAL) cells in regard to the available *PEI F25-LMW/siRNA polyplexes in the lung* (Fig. 2).*Thereby allowing for a direct comparison to results of a former study by* Semmler-Behnke et al. [3]

## 2 Experimental design, materials and methods

PEI F25-LMW/^32^P-siRNA polyplexes and non-complexed ^32^P-siRNA were prepared as fully described in [1]. Either non-complexed siRNA or PEI F25-LMW/^32^P-siRNA polyplexes were intratracheally instilled to groups of animals. At each time point (1 h, 3 h and 7 h), a minimum of three animals were exsanguinated, all organs, blood and carcass were collected. A bronchoalveolar lavage (BAL) was performed. BAL suspension was centrifuged in order to distinguish between BAL cells and BAL fluid. Samples were treated with nitric acid (50% v/v; one ml per mg sample weight) to obtain homogenous solutions for an analysis via LSC (liquid scintillation counting; beta-radio analysis). Values were corrected for background radiation and blood content within each organ. Either the sum of all animal samples or the sum of all lung-related samples served as denominator for the percentage calculation. All steps are described in detail in [1].
